# A Study of the Relationship Between Uric Acid and Substantia Nigra Brain Connectivity in Patients With REM Sleep Behavior Disorder and Parkinson's Disease

**DOI:** 10.3389/fneur.2020.00815

**Published:** 2020-08-05

**Authors:** Timothy M. Ellmore, Jessika Suescun, Richard J. Castriotta, Mya C. Schiess

**Affiliations:** ^1^Department of Psychology, The City College of New York, New York, NY, United States; ^2^Department of Neurology, The University of Texas McGovern Medical School at Houston, Houston, TX, United States; ^3^Department of Clinical Medicine, Keck School of Medicine of University of Southern California, Los Angeles, CA, United States

**Keywords:** uric acid, REM sleep behavior disorder, parkinson's disease, resting state, functional connectivity

## Abstract

Low levels of the natural antioxidant uric acid (UA) and the presence of REM sleep behavior disorder (RBD) are both associated with an increased likelihood of developing Parkinson's disease (PD). RBD and PD are also accompanied by basal ganglia dysfunction including decreased nigrostriatal and nigrocortical resting state functional connectivity. Despite these independent findings, the relationship between UA and substantia nigra (SN) functional connectivity remains unknown. In the present study, voxelwise analysis of covariance was used in a cross-sectional design to explore the relationship between UA and whole-brain SN functional connectivity using the eyes-open resting state fMRI method in controls without RBD, patients with idiopathic RBD, and PD patients with and without RBD. The results showed that controls exhibited a positive relationship between UA and SN functional connectivity with left lingual gyrus. The positive relationship was reduced in patients with RBD and PD with RBD, and the relationship was found to be negative in PD patients. These results are the first to show differential relationships between UA and SN functional connectivity among controls, prodromal, and diagnosed PD patients in a ventral occipital region previously documented to be metabolically and structurally altered in RBD and PD. More investigation, including replication in longitudinal designs with larger samples, is needed to understand the pathophysiological significance of these changes.

## Introduction

Parkinson's disease (PD) is a chronic, progressive neurologic disease characterized by motor deficits that include tremor at rest, rigidity, slowing of movement, and postural instability. PD pathology includes extensive loss of brain dopaminergic neurons, which occurs before the emergence of gross neurologic deficits, as well as the presence of Lewy body eosinophilic inclusion within neurons. Etiologic factors include a role for aging (1), a role for environmental factors (2, 3) including herbicide/pesticide exposure, and specific disease-causing genetic mutations, most notably in the α-synuclein (4), and the parkin (5) genes. Pathogenic mechanisms proposed to underlie the neuronal degeneration in PD include free radicals, deficits in energy metabolism, specifically abnormalities of iron metabolism and mitochondrial complex I, programmed cell death, and protein aggregation. The free radical-mediated injury theory, which is also referred to as the oxidant stress hypothesis, is arguably the leading explanation for pathogenesis due most notably to the fact that the major degradative pathway for dopamine is its oxidative deamination by monoamine oxidase A and B resulting in highly reactive hydroxyl radicals (6). While the oxidant stress hypothesis receives indirect support from numerous lines of evidence which have been extensively reviewed (7, 8), it remains only a hypothesis with shortcomings that include that aspects of the hypothesis that are dependent on catecholamine metabolism are not relevant to degeneration of structures like the nucleus basalis, which is cholinergic, and which also degenerate in PD. Nevertheless, since oxidative stress appears to play a role in the pathogenesis of Parkinson's disease (PD) (9, 10), questions remain about the role of antioxidants in PD and especially during the prodromal stage.

Uric acid (UA) is the end product of purine metabolism, and it accounts for most of the antioxidant capacity in human blood (11). Higher serum UA levels are associated with lower risk of developing PD in the general population (12, 13) and slower disease progression in the PD population (14). The presumptive neuroprotective action of UA includes suppression of oxygen radical accumulation, stabilization of calcium homeostasis, preservation of mitochondrial function, chelation of iron and blocking of iron dependent oxidation reactions, and the slowing of the dopamine auto-oxidation rate in the caudate and SN of PD patients (15–19).

A notable characteristic of UA's influence in PD is sex differences (20). In men only, UA was the first biomarker shown to be consistently associated with lowering risk of developing PD (12, 21–23) by 33% (24) as well as changing disease prognosis in males with PD (25–29). A link between UA and the risk of development or progression of PD in women is weaker possibly due to biological differences in interactions between sex-specific hormones and UA. This occurs independently of factors including age, smoking, obesity, hypertension, thiazide use, and caffeine consumption, all of which have been associated with PD and uricemia (30, 31).

A major pathological signature of PD is cell death in the SN (32–37). Previous research also indicates reduced SN functional connectivity and basal ganglia dysfunction in rapid-eye-movement sleep behavior disorder (RBD) (38, 39). RBD is a parasomnia that is associated with an increased likelihood of developing either PD or another alpha-synuclein neurodegenerative disorder (40, 41). Identifying the neural changes accompanying RBD has become a research priority for developing objective markers of early diagnosis (42–44). Neuroimaging studies show that RBD is associated with altered striatal dopaminergic innervation (45, 46), striatal volumetric differences (47), and reduced nigrostriatal and nigrocortical resting state functional connectivity (38). More recent neuroimaging studies including structural MRI, [18F]fluorodeoxyglucose PET, and fMRI resting state connectivity implicate posterior cortical regions, including lingual gyrus, in PD and RBD (37, 48–50). A large proportion of patients develop Parkinson's disease years after the diagnosis of their RBD (51, 52). The presence of polysomnography-proven RBD is a prodromal marker, with a positive likelihood ratio (LR+) of 130 and a negative likelihood ratio (LR–) of 0.65. Meanwhile, low plasma urate levels are a risk marker, with an LR+ of 1.8 (in men) and an LR– of 0.88 (in men) (53).

To our knowledge, no previous study has investigated the relationship between SN functional connectivity and levels of UA in RBD or PD patients. UA levels are directly reduced within the SN of Parkinson's patients (15), which adds to the rationale for investigating how connectivity of this structure covaries with UA levels. Studies using the horseradish peroxidase retrograde transport technique motivate investigation of SN connectivity with cortex because they show SN cells give rise to highly collateralized axons and innervate different regions of cortex, including cingulate cortices, prefrontal and suprarhinal cortex, and entorhinal cortex, as well as subcortical sites (54). To date, only one cross-sectional study has been reported investigating the role of UA in RBD (55), but no studies have investigated the relationship between levels of UA and SN functional connectivity in either RBD or PD. One hypothesis is that SN connectivity decreases in the prodromal phase of idiopathic RBD and further decreases by the time the early diagnosis of Parkinson's disease is made. Understanding how the relationship between UA and SN functional connectivity differs during the prodromal and clinically-defined stages of PD may inform the ongoing debate about how UA levels are associated with changes in neural connectivity. Therefore, the objective of the present study is to explore the relationship between UA levels and SN functional connectivity in males using the eyes-open resting state fMRI method. Resting state fMRI allows for measurement of intrinsic neuronal fluctuations to identify markers of prodromal neurodegeneration (42). We used whole-brain voxelwise ANCOVA and a cross-sectional design to test the prediction that SN functional connectivity exhibits a positive relationship with UA in controls, and that this positive relationship decreases in RBD patients and decreases further in PD patients.

## Materials and Methods

### Experimental Design

A total of 66 participants were recruited from the sleep clinic or movement disorders clinic or referred to our study from its entry at www.clinicaltrials.gov (NCT00817726). All participants provided written informed consent under a study protocol (HSC-MS-08-0147) that was approved by the UTHealth Institutional Review Board. The study was conducted in accordance to the International Conference on Harmonization Good Clinical Practice Guideline and the principles of the Declaration of Helsinki. Inclusion criteria were men aged 35–75 years old who did not have an unstable medical condition, and met criteria for one of the study groups including an early-to-moderate PD group, an idiopathic RBD group, and a control group. For all participants in the study, we asked about first- and second-degree family members with PD or any other neurodegenerative disorder, but this information was not used as an exclusion criterion for RBD or PD. Each was assigned to one of these three groups according to the inclusion criteria detailed in the next paragraphs and listed in Table 1. Included are 11 control subjects (CON), 32 patients with REM Sleep Behavior Disorder (RBD), and 23 patients with Parkinson's disease (PD). A total of 16 of the PD patients were confirmed to have RBD, denoted in this paper as PD (RBD+) and seven of the PD patients were confirmed not to have RBD, denoted as PD (RBD–).

**Table 1:** Participant demographics and clinical measures.

	**Control**	**RBD**	**PD**
Number of subjects	11	32	23
Age (yrs.), mean (SD)	54.82 (12.77)	57.47 (8.20)	61.17 (10.91)
DoD, mean (SD)	NA	3.13 (2.23)	5.89 (5.48)
RBD by polysomnography (*n*), %	0 (0.0)	32 (100)	16 (69.56)
Handedness	9 R, 2 L	28 R, 3 L, 1 LR	21 R, 2 L
UA (mg/dl), mean (SD)	5.60 (1.02)	5.19 (1.24)	4.81 (0.92)
MOCA, mean (SD)	28.36 (2.01)	27.44 (4.35)	27.23 (2.89)
UPSIT, mean (SD)	34.36 (6.04)	29.31 (7.55)	19.48 (6.77)
UPDRS-M, mean (SD)	0.64 (1.21)	2.16 (4.04)	25.22 (14.96)
H&Y, mean (SD)	0.00 (0.00)	0.00 (0.00)	1.91 (0.95)
Tremor-dominant/akinetic-rigid^*^	NA	NA	10 TD, 9 AR, 4 Mixed
Laterality of disease involvement	NA	NA	PD (RBD+): 7 R, 9 L PD (RBD-): 5 R, 2 L Total: 12 R, 11 L
Interfering medications	None	2, Thiazide	1, Thiazide

*DoD, duration of disease; MOCA, Montreal Cognitive Assessment; UPSIT, University of Pennsylvania Smell Identification Test; UPDRS, Unified Parkinson's Disease Rating Scale; H&Y, Hoehn and Yahr Staging*.

* *Sub-type was determined using Schiess et al. criteria. NA, Not Applicable*.

The diagnosis of PD was made based on the United Kingdom Brain Bank Diagnostic Criteria (56). Patients with parkinsonian symptoms due to atypical parkinsonism, vascular PD, or medicine/toxin-induced parkinsonism were excluded. No genetic tests were administered to identify genetic forms of PD. However, young-onset PD was excluded, which has a higher genetic prevalence. To exclude advanced disease, we used the Hoehn and Yahr disability scale (57) with a cutoff of ≤ 3.5 in the off-medicine state. Twelve of the 23 PD patients had right lateralized disease involvement, while 11 of the 23 patients had left lateralized disease involvement. Of the 16 patients with PD (RBD+), seven were right lateralized and nine were left lateralized. Of the seven patients with PD (RBD–), five were right lateralized and two were left lateralized.

The RBD group diagnostic criteria was based on the American Academy of Sleep Medicine (AASM) (58). Nocturnal video-polysomnography (NPSG) was performed in an AASM-accredited sleep disorders center, with a minimum of 10% REM sleep recorded and at least 10% of REM epochs documented to be without atonia. The criteria for the control group included individuals who have no personal history or primary family history of PD or neurodegenerative disease, and no history of dream enactment or REM sleep without atonia. All subjects met the above inclusion criteria and were matched to members of the PD or RBD groups in age (± 3 years). All controls underwent a PSG to rule out the presence of RBD. The PD patients also underwent a PSG to determine whether they were PD (RBD+) or PD (RBD–).

All individuals underwent clinical, behavioral, and brain imaging in the off-medicine state defined as no PD medicines for at least 12 h before the assessment the night before. The clinical assessment included a Montreal Cognitive Assessment Test (MoCA), and Unified Parkinson's Disease Rating Scale (UPDRS) evaluation with I-IV subscales. Medications like thiazide diuretics, loop diuretics, allopurinol, colchicine, febuxostat among others could interfere with UA levels. None of the controls used interfering medications, and only two patients with RBD and one patient with PD used thiazide medication that could impact UA levels (Table 1).

### Uric Acid

Serum UA concentration was measured in an early morning blood sample drawn from a peripheral vein by using a uricase colorimetric method on non-fasting blood. A one-way analysis of variance (ANOVA) with one factor, group, with four levels of CON, RBD, PD (RBD+), and PD (RBD–) was performed on the UA measurements using Prism 8 for macOS Version 8.3.

### Magnetic Resonance Imaging

Each participant underwent magnetic resonance imaging (MRI) using a Philips 3T scanner (Philips Medical Systems, Bothell, WA). Since dopaminergic medications influence the functional MRI signal of the task and rest state (59, 60), patients with PD were scanned in the off-medication state. The structural images acquired included a T1-weighted magnetization-prepared rapid acquisition turbo field echo sequence (repetition time/echo time [TR/TE] = 8.4/3.9 ms; flip angle = 8 degrees; matrix size = 256 × 256; field of view = 240 mm; slice thickness = 1.0 mm, sagittal acquisition. The functional images acquired included a whole-brain echo-planar imaging (EPI) run sensitive to BOLD contrast (TE = 30 ms; flip angle = 90 degrees; 2 s TR; 150 dynamics; 2.75 × 2.75 × 3.5 mm voxel resolution) which was acquired while participants were instructed to rest while remaining still and fixating a white cross hair displayed on a black background during the functional acquisition.

### Image Analysis

Image processing was performed with the Analysis of Functional Neuroimages (AFNI) (61). AFNI's python script afni_proc.py was used to process each participant's structural and functional MRI using the “example 11” resting state analysis procedure (https://afni.nimh.nih.gov/pub/dist/doc/program_help/afni_proc.py.html). The steps included in this analysis were (1) Despiking: the shrinking of any large spikes in the fMRI time series, (2) time shifting: the correction of slice timing differences, (3) Aligning: determining the alignment between the fMRI time series and anatomical T1, (4) Spatial normalization: determining the alignment between the anatomical T1 and a template brain, and (5) Censoring and regression: removing timepoints due to excessive motion and regressing out from the censored time series the contributions to signal from the ventricles and local white matter. We used a relatively conservative threshold to censor TR pairs where the Euclidean Norm of the motion derivative exceeds 0.3. The proportion of volumes censored in each of the four groups was computed [CON = 0.199 (±0.162), RBD = 0.215 (±0.241), PD (RBD+) = 0.179 (±0.214), PD (RBD–) = 0.082 (±0.108)] and did not differ significantly among the groups [*F*_(3, 64)_ = 0.7449, *p* = 0.5297]. For volumes that were not censored, the computed motion regressors were regressed out as covariates of no interest. We used the recommended default -KILL option where the censored timepoints were removed rather than filled with zeros or by values interpolated from neighboring non-censored timepoint values.

Resting state functional connectivity was estimated in each subject using an average fMRI time series accumulated in voxels representing left and right SN. A mask representing left and right SN was built using the multi-contrast PD25 atlas (62) using the following steps. First, the PD25 T1 MPRAGE volume with 1 mm resolution was co-registered to each subject's native-space skull-stripped T1 anatomical volume using a non-linear warping algorithm in AFNI. The segmented left and right SN volumes of the PD25 atlas were warped into the native-space of the T1 anatomical by applying the non-linear warp transformation. Then the transform from the native T1 anatomical space to the TT_N27 brain template that was computed in the *afni_proc.py* was applied to the segmented left and right SN volumes of the PD25 atlas, which resulted in segmented left and right SN masks for each subject in Talairach space. The SN masks in Talairach space were averaged and thresholded to create a single group mask representing the location of left and right SN. The thresholding level ensured that voxels in this mask represented complete overlap in the location of SN in at least 50% of the subjects so that the SN seed vectors for each subject were derived from the same voxel locations in standard space for the exploratory group analyses.

### Statistical Analysis

Group resting state analysis was conducted in AFNI. First, the *3dSetupGroupInCorr* command was used to build files of whole-brain masked residual error time series (niml ^*^.ertts) for the CON, RBD, PD (RBD+), and PD (RBD–) groups. Next, the *3dGroupInCorr* command was used to compute a standardized Z-score difference map of the slope of the inverse hyperbolic tangent of the correlation (i.e., Fisher transformation) of a seed vector with every voxel time series in the brain. The seed vector was made by averaging the time series of non-zero seed voxels in the bilateral SN mask. The output of the *3dGroupInCorr* command resulted in a standardized correlation coefficient (*zcorr*) volume for each subject in each of the four groups CON, RBD, PD (RBD+), and PD (RBD–). These volumes were next input to AFNI's *3dMVM* command (63), a group-analysis program that performs traditional analysis of covariance (ANCOVA). A between-subject factor *group* included four levels, CON, RBD, PD (RBD+), and PD (RBD–), and a quantitative covariate variable *uric acid* was included to produce F statistical maps representing the main effect of *group*, the main effect of *uric acid*, and the interaction between *group* and *uric acid*. An additional *post-hoc* analysis was performed using the same ANCOVA model but with the images for the 11 patients with left disease onset [2 PD (RBD–) and 9 PD (RBD+)] flipped about the x-axis using AFNI's *3dLRflip* command. The rationale for this analysis was that it would align for these 11 patients their diseased and non-diseased hemispheres with the 12 other patients [5 PD (RBD–) and 7 PD (RBD+)] with right disease onset whose images were not flipped.

Given the exploratory nature of these analyses, statistical tests with a height threshold of *p* < 0.05 and a cluster extent threshold (k) of 50 or greater were evaluated. A cluster-wise multiple comparison correction was computed using *3dClustSim* to estimate the probability of false positive (noise-only) clusters. *3dClustSim* is based on simulating the noise field that interferes with detection of the “true” signal in the dataset. To do this, *3dClustSim* needs statistics about the spatial smoothness of the noise. These estimates were computed for each subject using *3dFWHMx* with the -acf option, which computes the spatial autocorrelation of the data as a function of radius, then fits that to a model of the form *ACF(r)* = a^*^exp(–r^*^r/(2^*^b^*^b))+(1-a)^*^exp(–r/c). *3dFWHMx* output the 3 model parameters (a,b,c). An average of these parameters across all subjects were input to *3dClustSim* (ACF 0.65, 3.70, 9.77) resulting in a FWHM of 9.51 mm with 3D grid dimensions of 64 × 76 × 60 (2.5 × 2.5 × 2.5 mm^3^) and 91,631 voxels in the brain mask (31.40% of total voxels). *3dClustSim* determined given an uncorrected height threshold (pthr) of 0.05 that a cluster of size 358 voxels or greater would occur <5% by chance assuming first-nearest neighbor clustering (above threshold voxels cluster together if faces touch). In *3dClustSim* smoothing simulated data over a finite volume is known to introduce edge artifacts. To minimize this, extra-large padded simulated volumes were made before blurring (64 × 76 × 60 pads to 96 × 120 × 96), which were trimmed back down to the desired size before continuing with the thresholding and cluster-counting steps. Clusters of a size that survived the multiple comparisons correction are denoted in results tables. Given the exploratory nature of these analyses, clusters with a size that did not reach statistical significance after the multiple comparisons correction are also included in results tables as it has been pointed out that reporting of statistical results using arbitrary cluster-forming height thresholds and arbitrary minimum cluster size is not in itself problematic (64). These tables can be used to confirm replication of results in other exploratory analyses (65) and facilitate meta-analyses (66).

## Results

### Analysis of Uric Acid Levels

The lab reference range for UA levels at Houston's Memorial Hermann Hospital is 3.80 to 8.00 mg/dl. All CON subjects had UA levels (mean = 5.62, SD = 1.15, min = 4.10, max = 7.50, range = 3.40) within the lab reference range. A total of 29 of 32 RBD subjects had UA levels (mean = 5.19, SD = 1.24, min = 3.10, max = 8.00, range = 4.90) within the lab reference range, with three having UA levels below the reference minimum of 3.80 mg/dl. A total of 6 of 7 PD (RBD–) subjects had UA levels (mean = 5.16, SD = 1.22, min = 3.5, max = 7.20, range = 3.70) within the lab reference range with one having a UA level below the reference minimum of 3.80 mg/dl. A total of 13 of 16 PD (RBD+) subjects had UA levels (mean = 4.66, SD = 0.81, min = 2.80, max = 5.90, range = 3.10) within the lab reference range with three having UA levels below the reference minimum of 3.80 mg/dl.

**Figure 1:**
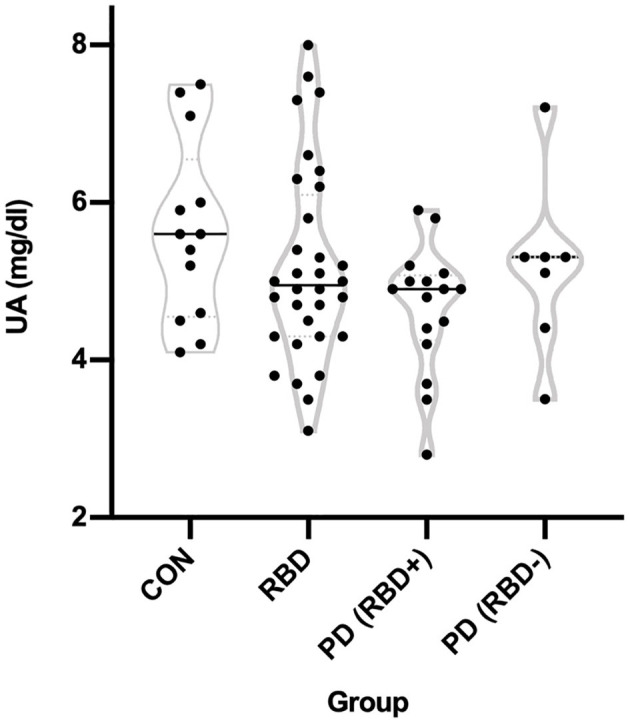
Distribution of uric acid levels in each group. Measured UA levels in the CON, RBD, PD (RBD+), and PD (RBD–) groups are shown using violin plots where the width of the distribution of points is proportionate to the number of points at a given Y value.

An ANOVA showed no main effect of group on UA levels [*F*_(3, 64)_ = 1.789, *p* = 0.158, *R*^2^ = 0.077]. The distribution of UA in each of the four groups is shown in Figure 1. *Post-hoc* analyses using Tukey's multiple comparisons test indicated no significant pairwise differences between the groups (Table 2). The largest difference was between CON and PD (RBD+), 5.623 vs. 4.662 mg/dl, but the mean difference of 0.961 was not significantly different (adjusted *p* = 0.110).

**Table 2:** Pairwise group comparisons of uric acid levels.

**Pairwise Comparison (Group 1 vs. Group 2)**	**Group 1 UA (mg/dl)**	**Group 2 UA (mg/dl)**	**Mean diff**	**SE of diff**	**95% C.I. of diff**	**Adjusted *p***
CON vs. RBD	5.62	5.19	0.44	0.37	−0.54 to 1.41	0.64
CON vs. PD (RBD+)	5.62	4.66	0.96	0.42	−0.14 to 2.07	0.11
CON vs. PD (RBD–)	5.62	5.16	0.47	0.53	−0.92 to 1.85	0.81
RBD vs. PD (RBD+)	5.19	4.66	0.53	0.34	−0.38 to 1.43	0.43
RBD vs. PD (RBD–)	5.19	5.16	0.03	0.47	−1.20 to 1.27	>0.99
PD (RBD+) vs. PD (RBD–)	4.66	5.16	–0.49	0.51	−1.84 to 0.85	0.76

*UA levels among the groups were compared using a 1-way ANOVA with group as factor and four levels of CON, RBD, PD (RBD+), and PD (RBD−). The main effect of group was not significant [F_(3, 64)_ = 1.79, p = 0.15]. Each pairwise comparison is shown with mean UA for each group, the group difference, the standard error of the group difference, the 95% confidence interval (C.I.) for the difference, and the adjusted p-value resulting from the Tukey's multiple comparisons test. The largest mean difference was between CON vs. PD (RBD+) with 0.96 higher UA for CON, but this difference did not reach significance (p = 0.11)*.

### Analysis of Substantia Nigra Functional Connectivity

**Figure 2:**
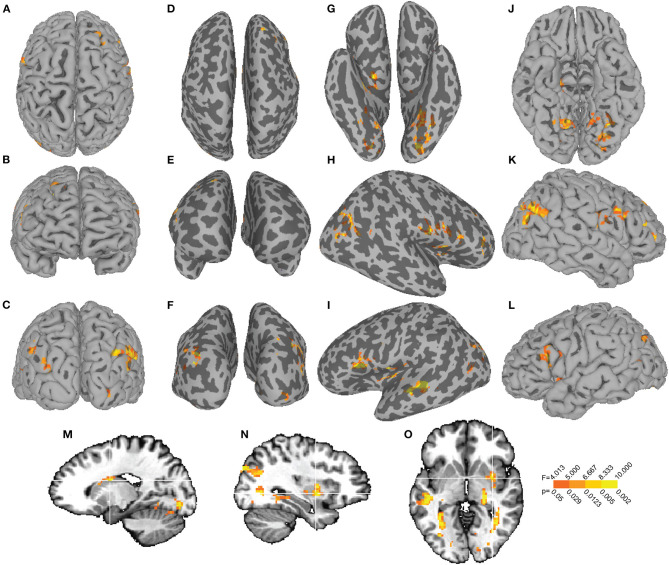
The main effect of uric acid on SN functional connectivity. The main effect of UA F-map is displayed on dorsal **(A)**, anterior **(B)**, and posterior **(C)** views of the pial surface with corresponding inflated representations **(D–F)**. Inflated views of the ventral **(H)**, right hemisphere **(I)**, and left hemisphere **(J)** cortex are displayed next to their pial representations **(K–M)**. Sagittal slices through left **(N)** and right **(O)** hemisphere and an axial slice **(P)** show main effects in left caudate (cluster 5, Table 3b) and right putamen (cluster 10, Table 3b).

Voxelwise ANCOVA identified eight brain clusters showing a main effect of group on SN functional connectivity (Figure 2). These clusters encompassed right nucleus accumbens, left superior temporal gyrus, right parahippocampal gyrus, left caudate, left cingulate gyrus, left superior frontal gyrus, right brainstem, and right cerebellum (Table 3a).

**Table 3:** Brain regions identified by ANCOVA.

**Cluster**	**Cluster Size**	**X**	**Y**	**Z**	**Brain Region**	**Peak (F, p)**
**a) Main effect: group**						
1	200	6	1	−12	R. Nucleus Accumbens	6.30,0.0009
2	73	−42	−42	11	L. Superior Temporal Gyrus	6.15, 0.001
3	72	25	8	−23	R. Parahippocampal Gyrus	7.72, 0.0002
4	68	−10	17	−5	L. Caudate	7.19, 0.0003
5	61	−17	24	29	L. Cingulate Gyrus	5.11, 0.003
6	60	−10	26	56	L. Superior Frontal Gyrus (BA 6)	7.55, 0.002
7	55	4	−30	−35	R. Brainstem	5.77, 0.001
8	52	28	−65	−35	R. Cerebellum (Crus 1)	6.32, 0.0009
**Cluster**	**Cluster Size**	**X**	**Y**	**Z**	**Brain Region**	**Peak F(F, p)**
**b) Main effect: UA**						
1	459^†^	−17	−86	−9	L. Lingual Gyrus (BA 18)	14.13, 0.0004
2	209	52	−73	34	R. Angular Gyrus	12.80, 0.0007
3	171	62	−12	20	R. Postcentral Gyrus	14.39, 0.0003
4	118	−53	19	16	L. Inferior Frontal Gyrus (BA 45)	20.24, 3.33e-05
5	118	−17	4	19	L. Caudate	10.74, 0.002
6	90	−44	−10	3	L. Insula	13.70, 0.0005
7	88	22	−15	−16	R. Hippocampus	10.34, 0.002
8	86	38	−58	−2	R. Inferior Temporal Gyrus	12.71, 0.0007
9	86	12	−60	−11	R. Cerebellum (Culmen)	12.03, 0.001
10	74	33	2	−6	R. Putamen	11.91, 0.001
11	66	22	42	37	R. Superior Frontal Gyrus (BA 8)	11.72, 0.001
12	65	−49	−26	−4	L. Superior Temporal Gyrus	15.39, 0.0002
13	64	49	−1	2	R. Insula	15.45, 0.0002
14	57	43	28	12	R. Inferior Frontal Gyrus (BA 45)	12.91, 0.0007
15	54	17	−91	−3	R. Lingual Gyrus	12.33, 0.0009
16	51	−26	−33	4	L. Thalamus	15.07, 0.0003
**Cluster**	**Cluster Size**	**X**	**Y**	**Z**	**Brain Region**	**Peak F**
**c) Interaction: group-by-UA**						
1	250^‡^	−23	−90	−20	L. Lingual Gyrus (BA 18)	8.33, 0.0001
2	215	28	35	34	R. Middle Frontal Gyrus (BA 8)	7.66, 0.0002
3	134	31	−19	50	R. Precentral Gyrus	7.19, 0.0003
4	89	−23	−28	−35	L. Cerebellum (IV-V)	9.70, 2.78e-05
5	85	−36	−88	14	L. Middle Occipital Gyrus (BA 19)	4.99, 0.004
6	80	−17	−9	14	L. Thalamus	10.01, 2.06e-05
7	79	15	−63	−10	R. Lingual Gyrus	6.89, 0.0005
8	74	−6	−69	13	L. Calcarine Gyrus	5.17, 0.003
9	72	1	−23	−38	R. Brainstem	10.56, 1.22e05
10	72	36	−32	16	R. Heschl's Gyrus	7.14, 0.0004
11	60	22	52	−9	R. Superior Frontal Gyrus (BA 10)	6.23, 0.001
12	59	−36	−31	8	L. Heschl's Gyrus	7.27, 0.003
13	56	17	58	10	R. Superior Frontal Gyrus (BA 10)	5.58, 0.002
14	56	−6	55	24	L. Superior Frontal Gyrus (BA 9)	7.24, 0.0003
15	50	31	−26	−4	R. Lentiform Nucleus	6.99, 0.0004

*The voxelwise ANCOVA analysis produced clusters for the main effect of group (a), the main effect of uric acid (b), and the group-by-uric acid interaction (c). For each of the three F maps, clusters exceeding a joint threshold (cluster height p < 0.05 & cluster size k ≥ 50 voxels) are listed in descending order by k along with the corresponding Montreal Neurological Institute x,y,z coordinate, labeled brain region with Brodmann Area (BA) where applicable, peak F value of the cluster and associated probability value. †Cluster exceeds threshold corrected for multiple comparisons. Cluster is a subset of cluster †in b*.

**Figure 3:**
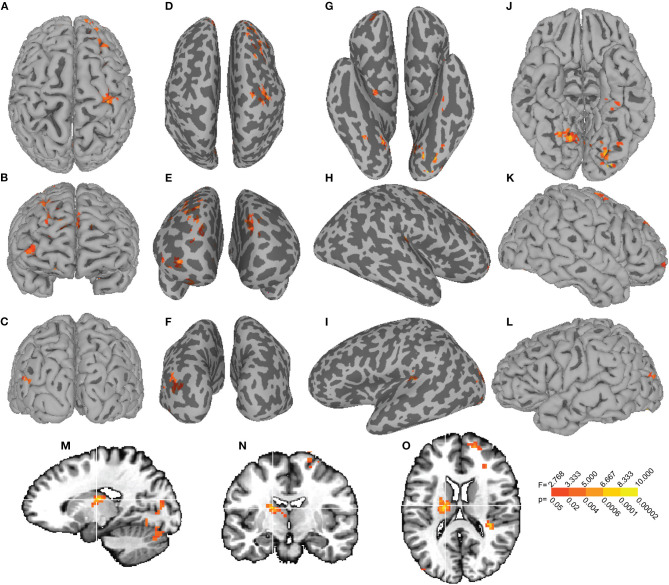
The group-by-uric acid interaction effect on SN functional connectivity. The group-by-UA interaction F-map is displayed on dorsal **(A)**, anterior **(B)**, and posterior **(C)** views of the pial surface with corresponding inflated representations **(D–F)**. Inflated views of the ventral **(H)**, right hemisphere **(I)**, and left hemisphere **(J)** cortex are displayed next to their pial representations **(K–M)**. A sagittal slice through left hemisphere **(N)** and coronal **(O)** and axial **(P)** slices show the interaction between group and UA on SN functional connectivity with the left thalamus (cluster 6, Table 3c).

Voxelwise ANCOVA identified 15 clusters showing a main effect of UA on SN functional connectivity (Figure 3). These clusters included left lingual gyrus, right angular gyrus, right postcentral gyrus, left inferior frontal gyrus, left caudate, left insula, right hippocampus, right inferior gyrus, right cerebellum and right putamen (complete list in Table 3b). The largest cluster, encompassing 459 voxels [peak *F*_(1, 58)_ = 14.13, *p* = 0.0004], survived the multiple comparison correction and was centered at MNI *x* = −17, *y* = −86, *z* = −9 in left lingual gyrus (BA 18).

Voxelwise ANCOVA also identified 15 clusters exhibiting an interaction between group and UA on SN functional connectivity. These clusters encompassed left lingual gyrus, right middle frontal gyrus, right precentral gyrus, left cerebellum, left middle occipital gyrus, left thalamus, right lingual gyrus, left calcarine gyrus, right brainstem, and right Heschl's gyrus (Table 3c). The left lingual gyrus cluster of size 250 [peak *F*_(3, 58)_ = 8.33, *p* = 0.0001] was centered at MNI *x* = −23, *y* = −90, *z* = −20 and encompassed a subset of the 459 voxel left lingual gyrus cluster identified in the main effect of UA F-map (cluster 1, Table 3b), which survived the multiple comparison correction. For the *post-hoc* ANCOVA in which the 11 patients with left disease onset were flipped, the previous largest cluster encompassing lingual gyrus (cluster 1, Table 3c) reflecting a Group-by-UA interaction increased in extent from 250 voxels to 416 voxels (cluster 1, Supplemental Table 1). A smaller cluster in the other hemisphere encompassing lingual gyrus (cluster 7, Table 3c) increased in extent from 79 voxels to 108 voxels (cluster 5, Supplemental Table 1).

**Figure 4:**
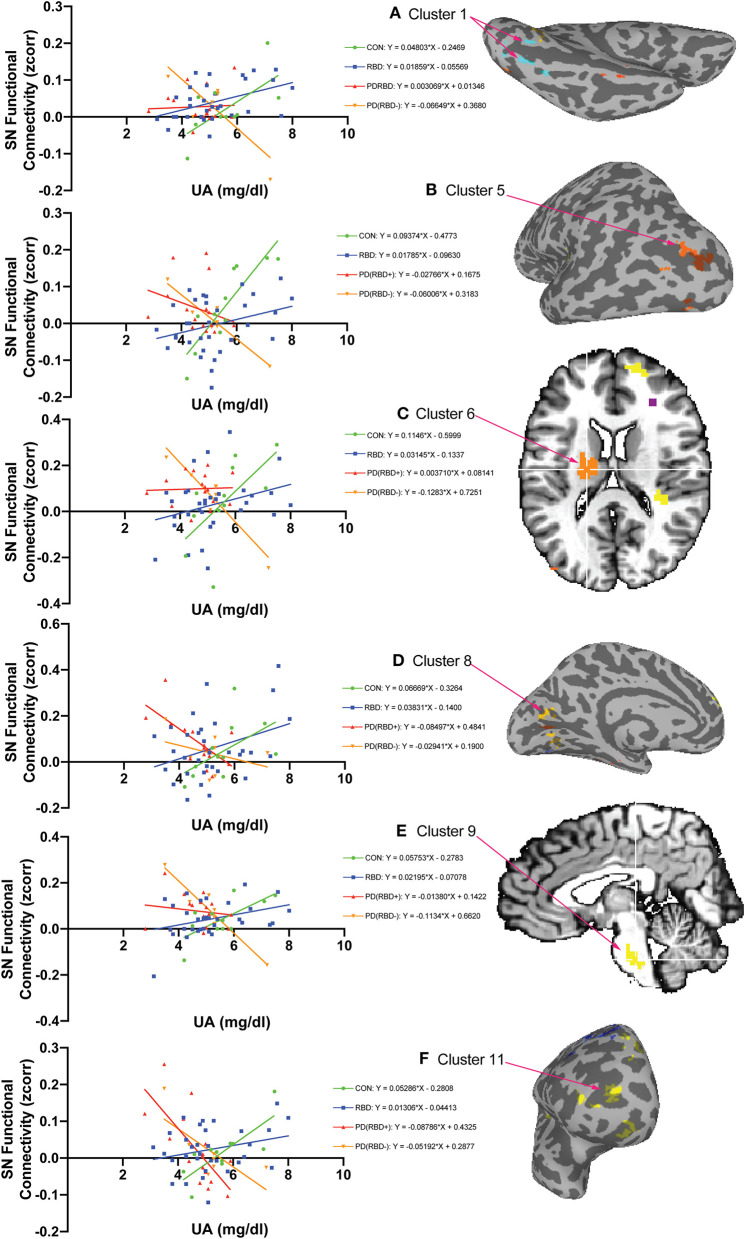
The group-by-uric acid interaction reveals six brain regions with decreasing SN functional connectivity as a function of uric acid from controls to PD with RBD in between. Of the 15 clusters identified in the ANCOVA interaction F-map, six exhibited a relationship in which CON subjects showed the highest positive regional slope [slope *m* = 0.04803 in **(A)** Cluster 1, L. Lingual Gyrus BA 18], the RBD slope was lower [*m* = 0.01859 in **(A)** Cluster 1], the PDRBD slope was lower still (*m* = 0.003069 in **(A)** Cluster 1], and the PD slope was negative [*m* = −0.06649 in **(A)** Cluster 1]. This ordered pattern of decreasing slopes across these groups is similar in the other clusters: cluster 5 **(B)**, cluster 6 **(C)**, cluster 8 **(D)**, cluster 9 **(E)**, and cluster 11 **(F)**. Slopes are computed using the average of voxels in each cluster in each subject. Cluster numbers are the same as in Table 3C. The color of cluster voxels in the inflated and orthogonal views is arbitrary.

Of the 15 clusters identified in the ANCOVA interaction F-map, six clusters (Figures 4A–F) exhibited a relationship in which CON individuals had the highest positive slope, with RBD patients showing a less positive slope, and the PD (RBD+) and PD (RBD–) patients having slopes lower than the RBD subjects. This graded decrease in slopes across the groups was evident in the left lingual gyrus cluster 1 (Figure 4A) in which the CON slope was 0.04803 [*F*_(1, 9)_ = 6.438, *p* = 0.0318], the RBD slope was a less positive 0.01859 [*F*_(1, 30)_ = 7.705, *p* = 0.0094], the PD (RBD+) slope was near flat at 0.003069 [*F*_(1, 14)_ = 0.04061, *p* = 0.8432] and the PD (RBD–) slope was negative at −0.06649 [*F*_(1, 5)_ = 9.517, *p* = 0.0273]. For the *post-hoc* ANCOVA in which the 11 patients with left disease onset were flipped and for which the cluster encompassing lingual gyrus increased in extent, the slopes exhibited a similar pattern with CON = 0.03763 [*F*_(1, 9)_ = 6.442, *p* = 0.0318], RBD = 0.01592 [*F*_(1, 30)_ = 7.856, *p* = 0.0088], PD (RBD+) = 0.00797 [*F*_(1, 14)_ = 0.5477, *p* = 0.4715], but with the biggest difference being a more significantly negative slope for PD (RBD–) = −0.0688 [*F*_(1, 5)_ = 16.10, *p* = 0.0102] (Supplemental Figure 1).

Of the 15 clusters identified in the ANCOVA interaction F-map, four clusters exhibited a relationship in which RBD patients had a positive slope while the CON individuals had negative or flat slopes. This relationship was evident in the right middle frontal gyrus cluster two (Supplemental Figure 2). In this right middle frontal gyrus cluster RBD subjects had a positive slope of 0.03231 [*F*_(1, 30)_ = 13.05, *p* = 0.0011], CON subjects had negative slope of −0.07525 [*F*_(1, 9)_ = 10.60, *p* = 0.0099], PD (RBD–) patients had a negative slope of −0.0446 [*F*_(1, 5)_ = 12.86, *p* = 0.0158], and PD (RBD+) patients had a nearly flat slope of −0.0215 [*F*_(1, 14)_ = 1.565, *p* = 0.2315].

Of the 15 clusters identified in the ANCOVA interaction F-map, four clusters exhibited a relationship in which the PD (RBD+) patients had a positive slope, while the CON subjects had a negative slope. This relationship was evident in the right precentral gyrus cluster 3 (Supplemental Figure 3). In this right precentral gyrus cluster PD (RBD+) patients had a positive slope of 0.09794 [*F*_(1, 14)_ = 14.53, *p* = 0.0019], CON subjects had a negative slope of −0.08411 [*F*_(1, 9)_ = 9.915, *p* = 0.0118], the PD (RBD–) subjects had a less negative slope of −0.03183 [*F*_(1, 5)_ = 3.603, *p* = 0.1161], and the RBD subjects had a flat slope of −3.496e-005 [*F*_(1, 30)_ = 6.411e006, *p* = 0.9980].

Of the 15 clusters identified in the ANCOVA interaction F-map, two exhibited a relationship in which the PD (RBD–) patients had a positive slope while the PD (RBD+) patients had a negative slope. This relationship was evident in the right lingual gyrus cluster 7 (Supplemental Figure 4). In this right lingual gyrus cluster the PD (RBD–) patients had a positive slope of 0.1741 [*F*_(1, 5)_ = 23.74, *p* = 0.0046], the PD (RBD+) patients had a negative slope of −0.03982 [*F*_(1, 14)_ = 4.775, *p* = 0.0464], the RBD patients had a nearly flat slope of 0.02382 [*F*_(1, 30)_ = 1.943, *p* = 0.1736] and the CON individuals also had a nearly flat slope of −0.01479 [*F*_(1, 9)_ = 0.2632, *p* = 0.6203].

## Discussion

The main novel finding of the present study is that a positive relationship was found between UA levels and SN resting state functional connectivity with posterior cortical regions in controls. Increased functional connectivity with higher UA levels in controls would appear consistent with the oxidant stress hypothesis, with levels of the antioxidant UA corresponding to stronger connectivity. But this relationship appears to break down in the patients: the positive relationship decreased in patients with RBD, and turned to a negative relationship in patients with PD. If the hypothesis that RBD is a prodromal state of PD, with idiopathic RBD a kind of intermediate state between controls and PD, then the pattern of slopes found with controls highest and positive, RBD in between, and PD lowest and negative may be consistent with more neurodegeneration from RBD to PD corresponding to reduced functional connectivity. This logic assumes that stronger functional connectivity is beneficial while reduced connectivity is detrimental, a relationship in neurodegenerative diseases that is supported by some data but remains a matter of debate especially in regards to functional compared to structural connectivity (67). Previous work has documented reduced resting state functional connectivity between SN and other basal ganglia regions as well as with posterior cortex with controls having highest connectivity, RBD patients in the middle and PD patients with lowest connectivity (38). The present study augments previous work by showing that a UA covariate is associated with changes in SN-posterior cortical connectivity. However, it is important to note that based on the present results nothing can be inferred about the neuroprotective role of UA in disease progression or connectivity. To address those questions, longitudinal imaging with UA levels taken at the same timepoints would need to be done to determine whether higher UA early after the diagnosis of RBD corresponded with a longer duration of time to convert (or never convert) to PD.

The physiological relevance of the altered functional connectivity between SN and lingual gyrus in RBD and PD found in the present study requires further investigation. A recent resting state fMRI reports reduced brain functional connectivity with a multiple posterior cortical regions in RBD patients in whom the connectivity changes correlated with mental processing slowness (50). Earlier studies using metabolic PET imaging (68) and corticometry (49) specifically implicate the lingual gyrus as one posterior cortical area showing reduced glucose tracer uptake and cortical thinning respectively in RBD vs. healthy controls. The lingual gyrus is typically associated with visual function and it has also been documented to show reduced gray matter along with superior parietal lobule in PD patients with visual hallucinations (69). If differences in the relationship between UA and SN functional connectivity in lingual gyrus can distinguish among disease states, that may be important for identifying prodromal biomarkers. However, a cross-sectional study like the present one is limited in this regard and it needs to be followed by future longitudinal imaging to track how functional connectivity changes with UA levels after the diagnosis of RBD and PD.

It is important not to draw firm conclusions regarding the hemispheric laterality of the present findings because patients present with asymmetric symptoms with some more compromised on the left and some more compromised on the right, which implicates opposite hemispheres in the disease pathophysiology. For our sample of PD patients with clinically significant asymmetric movement deficits, 12 had right disease onset and 11 had left disease onset. We attempted to address the issue of laterality in a supplemental *post-hoc* analysis in which the images of 11 patients with left disease onset as confirmed by clinical data were flipped. The rationale for this image flipping was that it would align their relatively more diseased and non-diseased hemispheres with the 12 other patients with opposite asymmetry whose images were not flipped. The prediction for this analysis was that it would increase magnitude of effects because without alignment of the diseased and non-diseased hemispheres, the effects would “average out” to some extent. The result of this subsequent *post-hoc* ANCOVA supported this idea as it showed the biggest increase in spatial extent for the largest cluster encompassing lingual gyrus in one hemisphere, and a smaller expansion in spatial extent of the other smaller cluster encompassing lingual gyrus in the opposite hemisphere. Inspection of regional slopes for the largest cluster revealed a similar pattern, but the negative slope for the PD (RBD–) group become more negative and more significant (Supplemental Figure 1).

While previous nigrocortical and cortico-cortical connectivity studies using resting state fMRI have reported left lateralized connectivity changes (38, 50, 70) consistent with left hemispheric predominance of nigrostriatal dysfunction (71), the laterality of connectivity results in the present study require further investigation. Laterality of disease onset is determined by neurologic exam and it is only possible to make these clinical determinations for the PD patients who show motor impairment. Controls and RBD subjects show no lateralized movement impairments, and in the absence of measurements from a gold-standard imaging technique like DaTscan (72) to quantify hemispheric differences in dopamine transporter levels, any lateralized functional connectivity differences in the present study should be interpreted with caution or not be interpreted at all without additional imaging evidence.

It has recently been reported using resting state fMRI that male *de novo* PD patients with higher UA levels had higher cortical functional connectivity in resting state networks including the dorsal attention network, executive control network, and default mode network, while female patients had lower functional connectivity regardless of UA level (73). These findings suggest that resting state networks might be closely and gender-specifically associated with the status of serum UA in *de novo* PD patients. Another study reports increased resting state connectivity between midbrain and cortex in PD (74). The organizational pattern of substantia connections with seven resting state functional networks has also been investigated to show that the medial portion of the SN compacta (mSNc) dominantly connects to limbic and visual cortex, while ventral SN (vSN) mainly connects with fronto-parietal and default mode networks (75). Widespread patterns of SN functional connectivity modulated by UA levels may also have a structural basis as diffusion tensor imaging reveals widespread structural connectivity of SN including primary motor cortex, somatosensory cortex, prefrontal cortex, caudate and putamen, globus pallidus, nucleus accumbens, temporal lobe, amygdala, pontine basis, occipital lobe, anterior and posterior cerebellum, corpus callosum, and external capsule (76).

Our main finding is that SN-posterior cortical resting state connectivity is positively associated with serum UA in male controls, and this relationship decreases in RBD and turns negative in PD. However, we also found a trend toward *increased* SN functional connectivity as a function of UA in frontal cortex and cerebellum in RBD patients (Supplemental Figure 2) and in cortex and lentiform nucleus in PD patients with and without RBD (Supplemental Figures 3, 4), but these results did not survive multiple comparisons correction. A limitation of the present study is relatively small sample sizes in the control and PD (RBD–) groups, which limits statistical power. Future studies utilizing larger sample sizes and more powerful longitudinal designs are needed investigate how different patterns of connectivity distinguish among controls, prodromal individuals, and diagnosed PD patients.

## Conclusion

We conclude that UA and SN functional connectivity among controls, a prodromal idiopathic RBD group, and PD patients with and without RBD is altered differentially in a ventral occipital region previously documented to be metabolically and structurally altered in RBD and PD. Replication in longitudinal designs with larger samples supplemented by dopaminergic imaging is needed to clarify the relevance of these patterns as biomarkers in prodromal Parkinson's disease.

## Data Availability Statement

The datasets generated for this study are available on request to the corresponding author.

## Ethics Statement

The studies involving human participants were reviewed and approved by Committee for the Protection of Human Subjects and The University of Texas Health Science Center at Houston. The patients/participants provided their written informed consent to participate in this study.

## Author Contributions

TE, JS, RC, and MS contributed equally to study conception, design, data collection, edited the paper, and read the final draft. TE and JS analyzed the data. TE wrote the first draft of the paper. All authors contributed to the article and approved the submitted version.

## Conflict of Interest

The authors declare that the research was conducted in the absence of any commercial or financial relationships that could be construed as a potential conflict of interest.

## Supplementary Material

Supplemental Figure 1 The group-by-uric acid interaction reveals positive SN functional connectivity as a function of uric acid in RBD in four brain regions. Of the 15 clusters identified in the ANCOVA interaction F-map, four exhibited a relationship in which RBD subjects had a positive regional slope (slope *m* = 0.03231 in panel a Cluster 2, R. Middle Frontal Gyrus BA 8), while CON subjects had negative or flat slope (*m* = −0.07525 in panel a Cluster 2). Slopes are computed using the average of voxels in each cluster in each subject. Cluster numbers are the same as in Table 3c. The color of cluster voxels in the inflated and orthogonal slice views in this figure is arbitrary.

Supplemental Figure 2 The group-by-uric acid interaction reveals positive SN functional connectivity as a function of uric acid in PD (RBD+) in four brain regions. Of the 15 clusters identified in the ANCOVA interaction F-map, four exhibited a relationship in which PD (RBD+) patients had a positive regional slope (slope *m* = 0.09794 in panel a Cluster 3, R. Precentral Gyrus), while the slope for CON subjects was negative (*m* = −0.08411 in panel a Cluster 3). Slopes are computed using the average of voxels in each cluster in each subject. Cluster numbers are the same as in Table 3c. The color of cluster voxels in the inflated and orthogonal views is arbitrary.

Supplemental Figure 3 The group-by-uric acid interaction reveals opposite relationships between SN functional connectivity as a function of uric acid in PD (RBD+) and PD (RBD–) in two brain regions. Of the 15 clusters identified in the ANCOVA interaction F-map, two exhibited a relationship in which the PD (RBD+) patients had a negative regional slope (slope *m* = −0.03982 in panel a Cluster 7, R. Lingual Gyrus), while the slope for PD patients without RBD were positive (*m* = 0.1741 in panel a Cluster 7). Slopes are computed using the average of voxels in each cluster in each subject. Cluster numbers are the same as in Table 3c. The color of cluster voxels in the inflated and orthogonal views is arbitrary.

Supplemental Figure 4 Group-by-uric acid regional interaction for the largest cluster encompassing lingual gyrus in the *post-hoc* left disease onset image flip analysis. In the *post-hoc* ANCOVA with image flipping for left disease onset patients, the largest cluster encompassing lingual gyrus from the original analysis (cluster 1, Figure 4) exhibited a similar pattern of slopes with the biggest difference being a more negative and significant slope for the PD (RBD–) group. Slopes are computed using the average of voxels in each cluster in each subject.

Supplemental Table 1 Brain regions with a group-by-uric acid interaction in the *post-hoc* analysis with image flipping for the left disease onset patients. Regions exceeding a joint threshold (cluster height *p* < 0.05 & cluster size k≥50 voxels) are listed in descending order by k along with the corresponding Montreal Neurological Institute x,y,z coordinate, labeled brain region with Brodmann Area (BA) where applicable, peak F value of the cluster and associated probability value. ^†^Cluster exceeds threshold corrected for multiple comparisons. ^†^Cluster is a subset of cluster ^†^in Table 3b.
