# Supplementary figures and images for: A Study of the Relationship Between Uric Acid and Substantia Nigra Brain Connectivity in Patients With REM Sleep Behavior Disorder and Parkinson's Disease
Source: Front Neurol. 2020 Aug 5;11:815. doi: 10.3389/fneur.2020.00815 (PMC7419698; doi:10.3389/fneur.2020.00815)

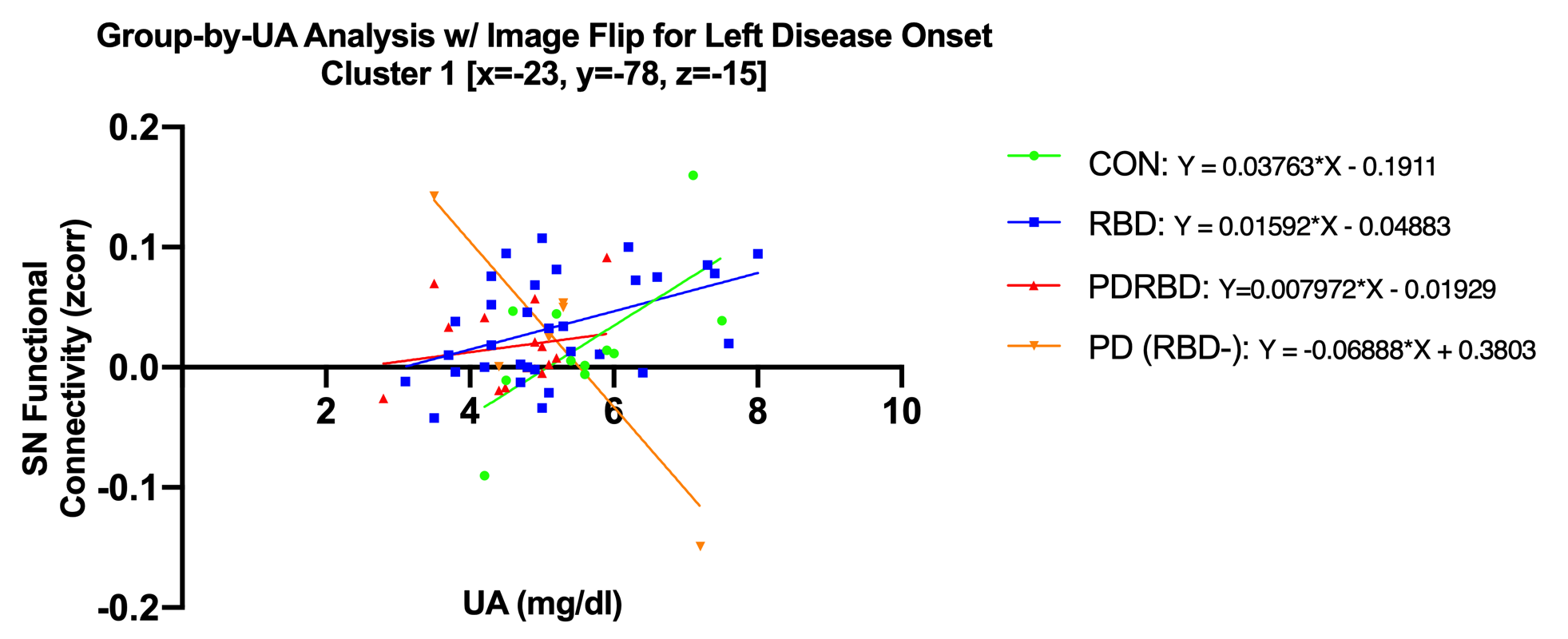

Supplement: Supplemental Figure 1 — The group-by-uric acid interaction reveals positive SN functional connectivity as a function of uric acid in RBD in four brain regions. Of the 15 clusters identified in the ANCOVA interaction F-map, four exhibited a relationship in which RBD subjects had a positive regional slope (slope m = 0.03231 in panel a Cluster 2, R. Middle Frontal Gyrus BA 8), while CON subjects had negative or flat slope (m = −0.07525 in panel a Cluster 2). Slopes are computed using the average of voxels in each cluster in each subject. Cluster numbers are the same as in Table 3c. The color of cluster voxels in the inflated and orthogonal slice views in this figure is arbitrary. [file Image_1.tiff]

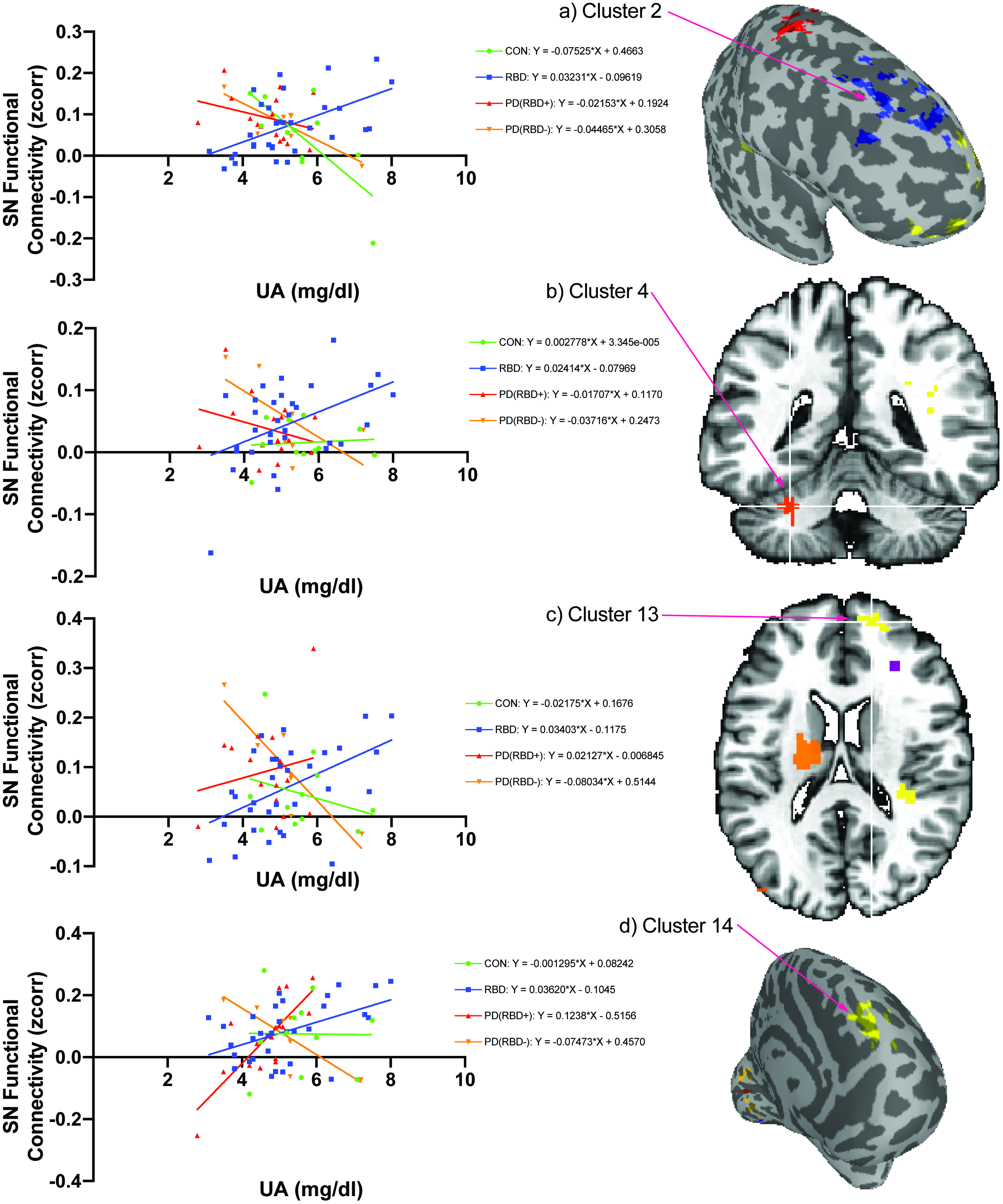

Supplement: Supplemental Figure 2 — The group-by-uric acid interaction reveals positive SN functional connectivity as a function of uric acid in PD (RBD+) in four brain regions. Of the 15 clusters identified in the ANCOVA interaction F-map, four exhibited a relationship in which PD (RBD+) patients had a positive regional slope (slope m = 0.09794 in panel a Cluster 3, R. Precentral Gyrus), while the slope for CON subjects was negative (m = −0.08411 in panel a Cluster 3). Slopes are computed using the average of voxels in each cluster in each subject. Cluster numbers are the same as in Table 3c. The color of cluster voxels in the inflated and orthogonal views is arbitrary. [file Image_2.tif]

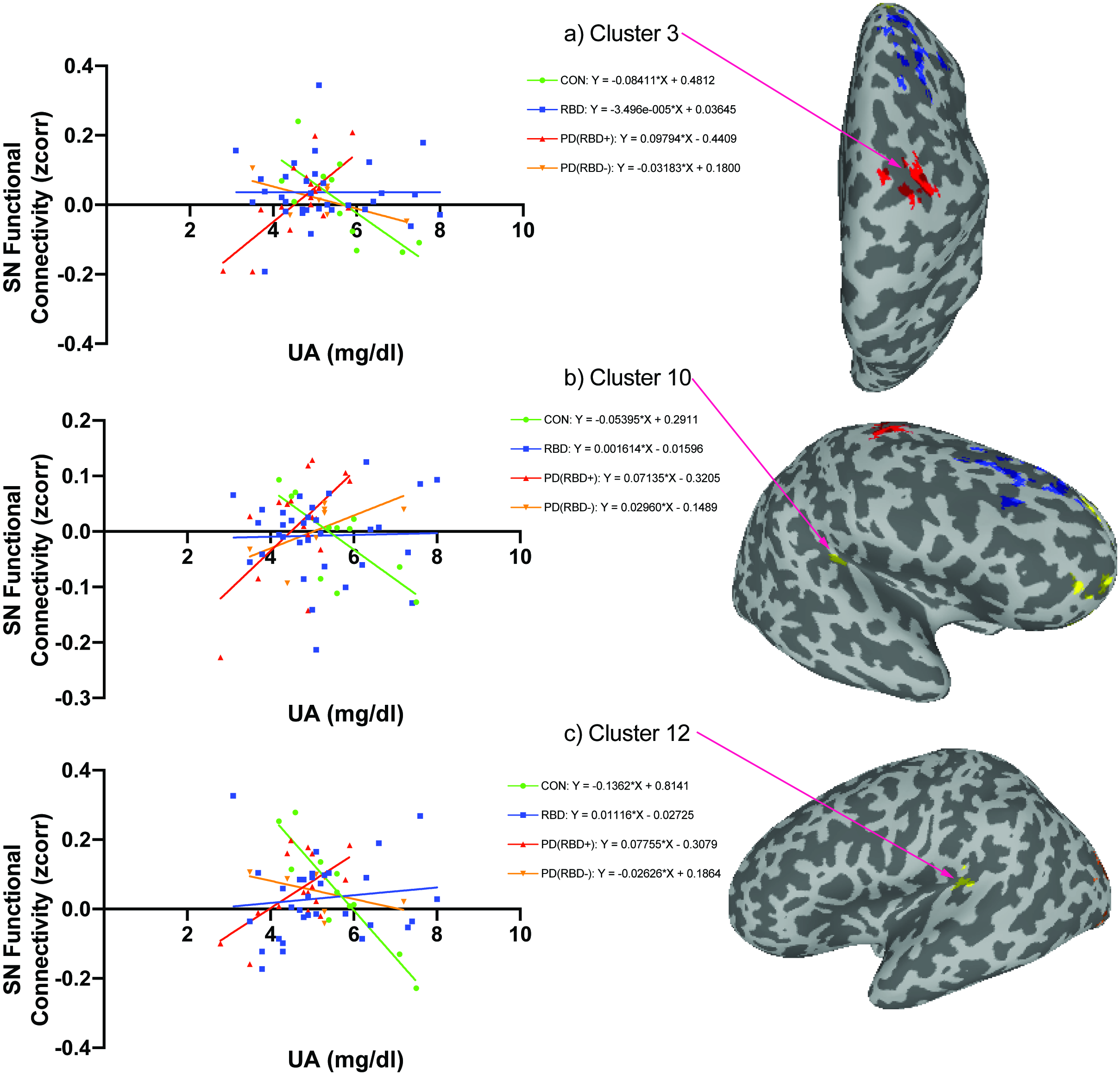

Supplement: Supplemental Figure 3 — The group-by-uric acid interaction reveals opposite relationships between SN functional connectivity as a function of uric acid in PD (RBD+) and PD (RBD–) in two brain regions. Of the 15 clusters identified in the ANCOVA interaction F-map, two exhibited a relationship in which the PD (RBD+) patients had a negative regional slope (slope m = −0.03982 in panel a Cluster 7, R. Lingual Gyrus), while the slope for PD patients without RBD were positive (m = 0.1741 in panel a Cluster 7). Slopes are computed using the average of voxels in each cluster in each subject. Cluster numbers are the same as in Table 3c. The color of cluster voxels in the inflated and orthogonal views is arbitrary. [file Image_3.tif]

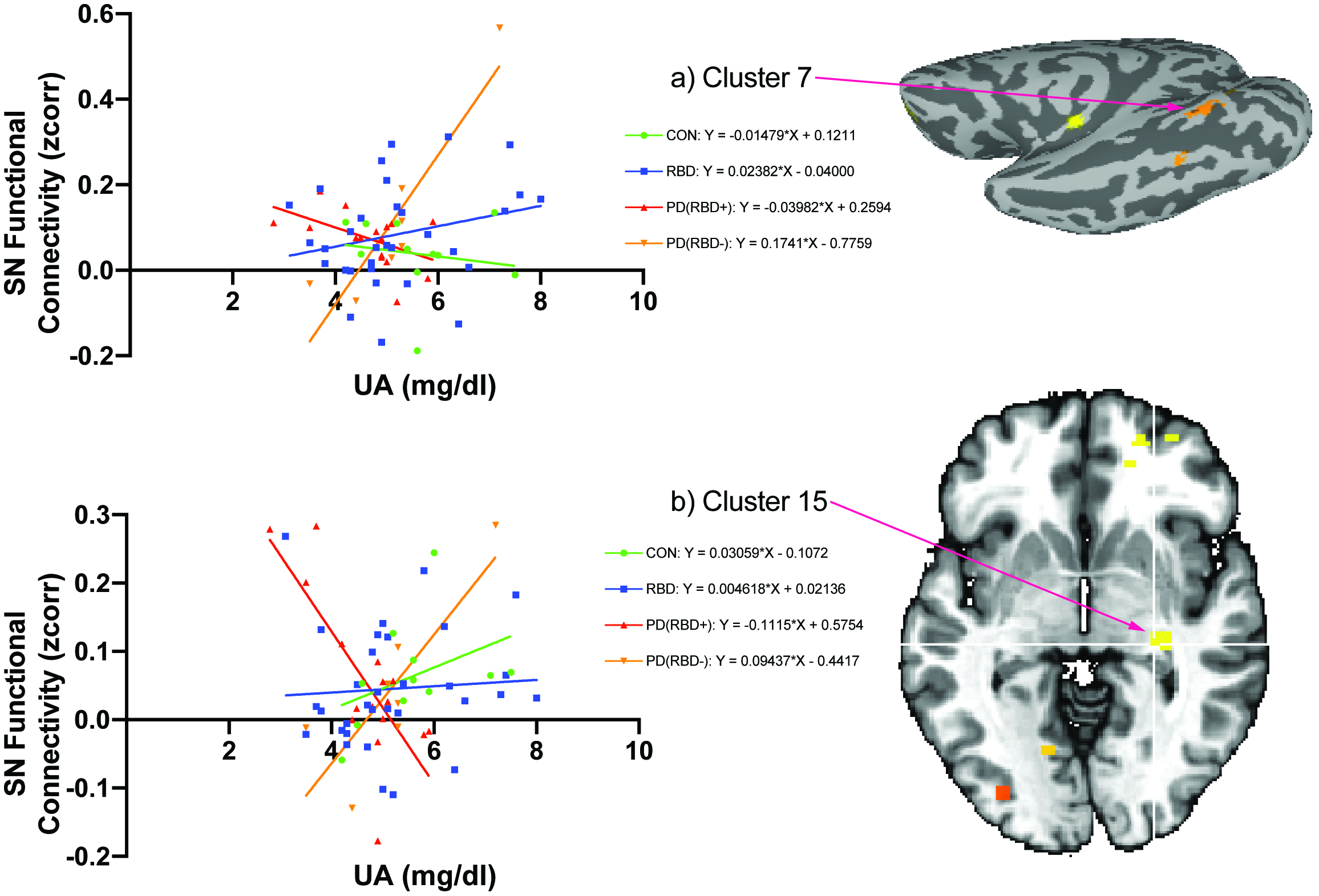

Supplement: Supplemental Figure 4 — Group-by-uric acid regional interaction for the largest cluster encompassing lingual gyrus in the post-hoc left disease onset image flip analysis. In the post-hoc ANCOVA with image flipping for left disease onset patients, the largest cluster encompassing lingual gyrus from the original analysis (cluster 1, Figure 4) exhibited a similar pattern of slopes with the biggest difference being a more negative and significant slope for the PD (RBD–) group. Slopes are computed using the average of voxels in each cluster in each subject. [file Image_4.tif]
