# Supplementary material for: A Study of the Relationship Between Uric Acid and Substantia Nigra Brain Connectivity in Patients With REM Sleep Behavior Disorder and Parkinson's Disease
Source: Front Neurol. 2020 Aug 5;11:815. doi: 10.3389/fneur.2020.00815 (PMC7419698; doi:10.3389/fneur.2020.00815)
Supplement: Supplemental Table 1 — Brain regions with a group-by-uric acid interaction in the post-hoc analysis with image flipping for the left disease onset patients. Regions exceeding a joint threshold (cluster height p < 0.05 & cluster size k≥50 voxels) are listed in descending order by k along with the corresponding Montreal Neurological Institute x,y,z coordinate, labeled brain region with Brodmann Area (BA) where applicable, peak F value of the cluster and associated probability value. †Cluster exceeds threshold corrected for multiple comparisons. †Cluster is a subset of cluster †in Table 3b. [file Table_1.DOCX]

| **Group-by-UA Post-Hoc Left Disease Onset Flip Analysis** | |  |  |  |  |  |
| --- | --- | --- | --- | --- | --- | --- |
| **Cluster** | **Cluster Size** | **X** | **Y** | **Z** | **Brain Region** | **Peak F** |
| 1 | 416†‡ | -23 | -78 | -15 | L. Lingual Gyrus (BA 18) | 19.71, 1.5986e-05 |
| 2 | 225 | -36 | -36 | 10 | L Superior Temporal Gyrus | 8.75, 0.002 |
| 3 | 155 | 6 | 59 | 19 | R. Superior Medial Gyrus | 9.18, 0.002 |
| 4 | 113 | 28 | 38 | 48 | R. Middle Frontal Gyrus | 7.51, 0.004 |
| 5 | 108 | 15 | -69 | -5 | R. Lingual Gyrus (BA 19) | 8.94, 0.002 |
| 6 | 95 | -20 | -47 | -42 | L. Cerebellum (IX) | 8.77, 0.002 |
| 7 | 82 | -15 | -74 | 13 | L. Calcarine Gyrus (BA 23) | 5.75, 0.01 |
| 8 | 76 | -23 | -3 | 14 | L. Putamen | 6.86, 0.006 |
| 9 | 67 | -20 | 49 | 28 | L. Middle Frontal Gyrus | 6.24, 0.008 |
| 10 | 63 | 1 | -23 | -38 | Brain Stem | 10.14, 0.001 |
| 11 | 57 | 59 | 32 | 0 | R. Inferior Frontal Gyrus (BA 45) | 7.37, 0.004 |
| 12 | 56 | 4 | 19 | 64 | R. Superior Frontal Gyrus (BA 6) | 5.61, 0.011 |
| 13 | 53 | -63 | 15 | 39 | R. Superior Frontal Gyrus (BA 10) | 9.23, 0.002 |

**Supplemental Table. Brain Regions with a Group-by-Uric Acid Interaction for the Post-Hoc Analysis with Image Flipping for the Left Disease Onset Patients.** Regions exceeding a joint threshold (cluster height p<0.05 & cluster size k>50 voxels) are listed in descending order by k along with the corresponding Montreal Neurological Institute x,y,z coordinate, labeled brain region with Brodmann Area (BA) where applicable, peak F value of the cluster and associated probability value. † = cluster exceeds threshold corrected for multiple comparisons. ‡ = cluster is a subset of cluster † in Table 3b.
